# Psychoactive substance use in patients diagnosed with attention-deficit/hyperactivity disorder: an exploratory study

**DOI:** 10.3389/fpsyt.2023.1184023

**Published:** 2023-07-11

**Authors:** Gniewko Więckiewicz, Iga Stokłosa, Maciej Stokłosa, Włodzimierz Więckiewicz, Piotr Gorczyca, Tomasz M. Gondek

**Affiliations:** ^1^Department of Psychiatry, Faculty of Medical Sciences in Zabrze, Medical University of Silesia, Katowice, Poland; ^2^Department of Prosthodontics, Faculty of Dentistry, Wroclaw Medical University, Wroclaw, Poland; ^3^Iter Psychology Practices, Wroclaw, Poland

**Keywords:** ADHD, psychoactive substance, psychoactive substance abuse, addiction, addiction psychiatry

## Abstract

**Introduction:**

Attention-deficit/hyperactivity disorder (ADHD) was originally treated as a neurodevelopmental disorder that occurs mainly in children and tends to diminish or disappear with age, but we now know that symptoms persist into adulthood in over 50% of ADHD patients. Undiagnosed individuals often turn to psychoactive substance to minimize the negative aspects of functioning and improve quality of life.

**Methods:**

The study was conducted online using random sampling through a Facebook group administered by physicians and targeted to patients diagnosed with ADHD. The study was naturalistic and exploratory, therefore no hypothesis was made. 438 correctly completed questionnaires were received. Analysis of the results showed that people with ADHD turn to psychoactive substances relatively frequently.

**Results:**

The most commonly used stimulants include alcohol, marijuana, 3,4-methylenedioxymethamphetamine (MDMA), amphetamine/methamphetamine, and psilocybin. In the study population, methylphenidate is the most commonly used drug among patients. After treatment with psychostimulants, the majority of respondents note a decrease in symptoms of hyperactivity disorder, especially in male patients.

**Conclusion:**

It is necessary to perform proper diagnostics and actively look for ADHD symptoms in patients who tend to use psychoactive substances.

## Introduction

Attention-deficit/hyperactivity disorder (ADHD) was originally considered a neurodevelopmental disorder that occurs mainly in children and subsides or disappears with age. However, it is now known that in a significant proportion of individuals with ADHD, symptoms persist into adulthood (1, 2). Symptoms diagnosed in childhood persist into adolescence in 60–85% of adolescents and between 2–46% of adults (3). The prevalence of the disorder varies between 2.5 and 4% in adults (3). According to ICD-11, ADHD is diagnosed when there is a persistent pattern of symptoms that include inattention, hyperactivity, and impulsivity. These symptoms must have started before the age of 12 and be more severe than what is typically expected for someone’s age and intellectual development. The symptoms should last for at least six months and have a significant negative impact on academic, occupational, or social functioning. The symptoms should also be present in multiple situations or settings, such as at home, school, work, or with friends and relatives. However, the specific symptoms may vary depending on the structure and demands of the setting. It’s important to note that the symptoms should not be caused by substances or medications affecting the central nervous system, withdrawal effects, a nervous system disorder, or be better explained by another mental disorder such as anxiety, fear-related disorders, or neurocognitive disorders like delirium (4).

Studies have shown that, in contrast to children, adults suffering from ADHD have fewer symptoms from the hyperactivity and impulsivity spectrum, while symptoms from the inattention group seem to predominate (5). In adults, additional symptoms include emotional lability, irritability, increased talkativeness, and rest problems, and difficulties in completing complex tasks, time management, and easy distractibility occur in the context of attention deficit (6). There are the following subtypes of ADHD: predominantly inattentive or predominantly hyperactive–impulsive and mixed. Each subtype may be associated with a predisposition to certain behaviors-the inattentive subtype may be associated with low self-esteem and negative performance in the workplace, while the hyperactive–impulsive subtype may result in a greater tendency toward aggression, risky driving behaviors, and more frequent peer rejection (7).

Other disorders may also coexist with attention deficit hyperactivity disorder; studies have shown that up to 80% of adults diagnosed with ADHD may be affected, with the most commonly diagnosed disorders being substance abuse, mood and anxiety disorders, and dissocial personality (8). Substance abuse problems affect 1 in 5 adults diagnosed with ADHD; moreover, these patients are significantly less likely to maintain abstinence from psychoactive substances, and they have lower remission rates while participating in drug rehab programs and require a longer duration to achieve a satisfactory outcome (9). Not only does ADHD result in an increased propensity to abuse alcohol, but the risk of developing an alcohol dependence syndrome has been shown to be as high as 43% in ADHD patients, compared to 3–11% of individuals in the non-ADHD population. Problems with alcohol abuse are more common in adults than in adolescents (10).

Psychostimulants, especially amphetamine derivatives or methylphenidate, are most commonly used to treat attention deficit hyperactivity disorder, whereas atomoxetine is used when there are existing contraindications to the use of psychostimulants or when it is poorly tolerated. For stimulant abuse, methylphenidate in the form of OROS is the preferred choice, while atomoxetine is preferred for alcohol abusers. The next treatment option described in the studies is bupropion, which is not approved by the Food and Drug Administration (FDA) for the treatment of ADHD (11). Of note, bupropion has shown benefits in trials for the treatment of ADHD in combination with addiction and depressive disorders (12). In a study examining the effects of treating ADHD with amphetamine derivatives, stimulant therapy was shown not to increase the risk of developing substance abuse in individuals; on the contrary, treatment appears to reduce the risk of SPA abuse disorders by approximately 50% (13). When a comparision was made between several medications – lisdexamfetamine, mixed amphetamine salts, modafinil, and methylphenidate – the first one, lisdexamfetamine, had the most promising efficacy, acceptability, and tolerability in patients with ADHD (14). Unfortunately, it is not available for a regular prescriber in Poland. It seems noteworthy that, although further research is needed on this topic, the available research suggests that pharmacological treatment of ADHD related mental disorders, especially when it begins earlier and lasts longer, seems to be associated with a reduction in the development of dependence on psychoactive substances (15). It is critical to expand awareness and attention to symptoms that may be associated with ADHD in adults because the consequences of untreated attention deficit hyperactivity disorder, although affecting individual patient functioning, are often marginalized by researchers because they assume that the disorder is due to the nature of the patient. Undiagnosed individuals often turn to stimulants to minimize the negative aspects of functioning and make life more enjoyable (16). It is also important because ADHD is linked to higher mortality rates, especially when diagnosed in adulthood. Comorbid conditions like oppositional defiant disorder, conduct disorder, and substance use disorder further increase the mortality risk. Even after accounting for these comorbidities, ADHD remains associated with excess mortality, particularly in females, with accidents being the leading cause of death in individuals with ADHD (17). The association documented to date between nonmedical use of psychoactive substances and ADHD justifies the need to expand knowledge in this area.

The aim of our study was to collect baseline demographic-epidemiological data on psychoactive substance use in patients diagnosed with ADHD and to determine whether adults struggling with ADHD have a greater predisposition to psychoactive substance use and whether and how treatment affects stimulant use.

## Materials and methods

The study was designed by psychiatrists and conducted in accordance with the Declaration of Helsinki and good clinical practice guidelines. Because the study was naturalistic, we did not conduct any pilot study. According to the Ethical Committee of Medical University of Silesia anonymous, internet surveys that do not allow to deanonymize respondents do not require approval. A separate questionnaire was created to survey the population, which included basic sociodemographic questions and several other questions related to diagnoses or treatments for attention deficit hyperactivity disorder, among other factors such as psychoactive substance of choice. The list of psychoactive substance was based on PolDrugs, the biggest Polish naturalistic epidemiological psychoactive users survey for psychoactive users (18). In addition, validated Polish versions of Alcohol Use Disorders Identification Test (AUDIT) and Drug Use Disorders Identification Test (DUDIT) questionnaires were used to ensure reliability of data on psychoactive substance use. AUDIT is a widely used screening tool designed to assess alcohol consumption patterns, alcohol-related problems, and alcohol dependence. The AUDIT scale consists of 10 questions that cover various aspects of alcohol use, including frequency, quantity, and consequences of drinking, providing a comprehensive evaluation of an individual’s alcohol-related behaviors. The AUDIT scale has demonstrated good reliability and validity. DUDIT is also a screening tool but specifically designed to assess drug use patterns, drug-related problems, and drug dependence. Comprising of 11 items, the DUDIT scale covers a range of drug-related behaviors, including frequency, quantity, and consequences of drug use, allowing for a comprehensive evaluation of an individual’s drug use patterns and related issues. The DUDIT scale, just as AUDIT scale, also has demonstrated good reliability and validity (19).

The survey was conducted online, using convenience sampling through an online Facebook group that was created for people who were interested in ADHD, managed by physicians. Facebook uses cookies to suggest it’s users certain groups, therefore if people tried to search for ADHD in their search engines, our group could be suggested by Facebook. The group was created about 15 months before the study was conducted, and the group had 5,609 members on the day we started collecting the data (physicians, other healthcare workers, patients diagnosed with ADHD, people interested in ADHD). The platform used to created the survey was Google Forms, an easy-to-use tool for creating anonymous online surveys. The researchers did not ask for email addresses or IP addresses, and all data collected were processed in accordance with the data protection regulations of the Republic of Poland’ and the European Union’. Because the survey was an internet one, the consent was also web based and the respondents could not move to the survey without accepting the terms. Because the survey’ is about the possession and use of often illegal psychoactive substances, anonymity was critical to obtaining truthful responses from respondents. Data were collected between October 13, 2022 and October 24, 2022.

Participation in the study was voluntary, patients were informed of the purpose of the study, and consent was required in the form of an affirmative response to the question “I am at least 18 years of age and consent to participate in this anonymous study” in order to exclude individuals who merely suspected ADHD in themselves, respondents were also required to confirm in the questionnaire that they had received an ADHD diagnosis from a physician. Only after agreeing to participate was it possible to take part in the survey. A total of 438 completed questionnaires were collected from people who declared having ADHD diagnosed by a physician. The survey was constructed in a way that did not allow user to submit incomplete data however we cannot estimate how many people resigned from filling the survey while completing the survey.

The study was an exploratory and naturalistic study, i.e., no research hypothesis was established and the inclusion criteria were limited to a correctly completed questionnaire, but only sought to investigate and characterize the phenomenon of psychoactive substance use and the effects of appropriately selected medications on the reduction of this behavior in a population of adults with attention deficit hyperactivity disorder.

## Statistics

Statistical analysis was performed using the STATISTICA 13.3 program (StatSoft, Cracow, Poland). The chi-square test was used for the comparison of qualitative variables.

For quantitative variables, the Mann–Whitney U test was used for the comparison of two independent groups, whereas the Kruskal-Wallis test was used for the comparison of multiple independent groups.

Spearman’s rank correlation was used to test the correlation of linear variables.

To evaluate the parameters affecting AUDIT and the DUDIT scale, we performed univariate and multivariate linear regression analysis. The significance level was set at *p* < 0.05.

## Results

### Sample characteristics

Four hundred thirty-eight correctly completed questionnaires were received. The characteristics of the study population are shown in Table 1. Due to the small group of respondents identifying as non-binary gender (4.6%), it was decided to conduct a comparative analysis of the two genders – M and *K. no* significant differences were found between the genders in terms of age, education, place of residence, type of ADHD, or past drug treatment. There is a statistically significant difference in the distribution of sexual orientations between the 2 sexes, consistent with the proportions observed in our other studies of university students (χ^2^ = 19.5; *p* < 0.005; chi-square test). Similarly, there is a difference in work situation between genders (χ^2^ = 19.5; *p* < 0.005; chi-square test).

**Table 1:** Study group characteristics.

		All	M	K	Other	*p* value M *vs* K
	*n*	438	88	330	20	
	%	100.0%	20.1%	75.3%	4.6%	
	Age	29.5 ± 7.0	29.2 ± 6.4	29.9 ± 7.2	25.5 ± 5.4	NS (M-W U test)
Sexual orientation	Heterosexual	64.8%	76.1%	65.2%	10.0%	*p* < 0.005
	Bisexual	23.3%	9.1%	25.8%	45.0%	
	Homosexual	7.1%	11.4%	5.8%	10.0%	
	Other	4.8%	3.4%	3.3%	35.0%	
Size of residence	Village	5.3%	4.5%	5.8%	0.0%	NS (χ)^2^
	<50 k	8.0%	9.1%	8.2%	0.0%	
	50–200 k	11.2%	13.6%	10.6%	10.0%	
	>200 k	75.6%	72.7%	75.5%	90.0%	
Education	Basic	0.2%	1.1%	0.0%	0.0%	NS (χ)^2^
	Middle School	1.8%	3.4%	1.2%	5.0%	
	Medium	35.6%	40.9%	33.0%	55.0%	
	Higher Bachelor’s degree	25.6%	25.0%	25.8%	25.0%	
	Master’s degree	33.8%	25.0%	37.3%	15.0%	
	Doctorate or higher degree/title	3.0%	4.5%	2.7%	0.0%	
Professional situation	Full-time employee	52.1%	54.5%	52.7%	30.0%	*p* < 0.005 (χ)^2^
	Student	20.1%	18.2%	18.8%	50.0%	
	Entrepreneur	16.2%	19.3%	16.1%	5.0%	
	Casual worker	7.5%	0.0%	9.4%	10.0%	
	Student	1.8%	5.7%	0.6%	5.0%	
	Unemployment	2.3%	2.3%	2.4%	0.0%	
Have you ever been treated for addiction?	No	94.1%	92.0%	94.2%	100.0%	NS (χ)^2^
	Yes	5.9%	8.0%	5.8%	0.0%	
Has the type of ADHD been determined?	Do not know/subtype not specified	25.8%	30.7%	24.8%	20.0%	NS (χ)^2^
	ADHD with predominantly attention deficit	34.9%	29.5%	36.1%	40.0%	
	Mixed type ADHD	37.9%	37.5%	38.2%	35.0%	
	ADHD with predominance of hyperactivity and impulsivity	1.4%	2.3%	0.9%	5.0%	
Age of ADHD diagnosis		28.5 ± 7.7	27.5 ± 7.5	29.0 ± 7.7	23.8 ± 5.8	*p* < 0.005 (KW test, post-ad-hoc^*^)

NS, non significant; M-W U test, Mann Whitney U Test; χ^2^, Chi-squared test; KW test, Kruskal-Wallis H test; ^*^Significant difference between other vs. M and other vs. K; NS M vs. K.

In *post hoc* analysis, a significant difference in age of ADHD diagnosis was found between respondents who self-identified as “other” gender and gender M or K (*p* < 0.005; Kruskal-Wallis test).

### Pharmacotherapy

The characteristics of pharmacotherapy used by the respondents are shown in Table 2.

**Table 2:** Characteristics of used pharmacotherapy among respondents.

		All	M	K	Other	*p* value M *vs* K (χ)^2^
	*n*	438	88	330	20	
	%	100%	20.10%	75.30%	4.60%	
Current medications						
Methylphenidate	Yes	75.11%	76.14%	74.85%	75.00%	NS
	No	24.89%	23.86%	25.15%	25.00%	
Atomoxetine	Yes	10.27%	11.36%	10.00%	10.00%	NS
	No	89.73%	88.64%	90.00%	90.00%	
Duloxetine	Yes	3.65%	2.27%	3.94%	5.00%	NS
	No	96.35%	97.73%	96.06%	95.00%	
Other	Yes	9.36%	13.64%	7.88%	15.00%	NS
	No	90.64%	86.36%	92.12%	85.00%	
How many medications are currently being used	None	12.80%	10.20%	13.30%	15.00%	NS
	In the process of diagnosis/matching	1.40%	1.10%	1.50%	0.00%	
	One	68.70%	68.20%	69.40%	60.00%	
	More than one	17.10%	20.50%	15.80%	25.00%	
Were the medications discontinued	No	95.43%	94.32%	95.76%	95.00%	
	Yes, no reason specified	1.37%	2.27%	1.21%	0.00%	
	Yes, because of side effects	3.20%	3.41%	3.03%	5.00%	
Have you noticed any improvement in ADHD symptoms after the current treatment regimen?	No	8.00%	2.30%	9.40%	10.00%	
	Rather no	9.40%	8.00%	9.40%	15.00%	
	Rather yes	32.00%	34.10%	32.10%	20.00%	
	Yes	50.70%	55.70%	49.10%	55.00%	
Did your psychiatrist talk to you about the psychoactive substance you were using BEFORE you started treatment?	No	24.40%	21.60%	25.20%	25.00%	
	Yes	75.60%	78.40%	74.80%	75.00%	
AFTER you started treatment, did your psychiatrist talk to you about the psychoactive substance you were using?	No	61.20%	56.80%	62.10%	65.00%	
	Yes	38.80%	43.20%	37.90%	35.00%	
Have you noticed a change in psychoactive substance use since starting treatment?	Yes, I am taking them less	34.50%	44.30%	31.80%	35.00%	
	No, I do not use psychoactive substance or their frequency of use has not changed	60.70%	47.70%	64.20%	60.00%	
	Yes, I am taking more of them	4.80%	8.00%	3.90%	5.00%	

NS, non significant; χ^2^, Chi-squared test.

The vast majority of respondents (69%) use only one medication, and the most common is methylphenidate (75%). We did not collect information about methylphenidate formula. No statistically significant differences were found between genders and medications taken. Only 3% of patients discontinued medication due to side effects.

A reduction in ADHD symptoms after treatment was noted in 83% of respondents, although the difference between genders is borderline and could prove statistically significant with a larger sample (*p* = 0.057 = NS; chi-square test).

Up to 35% of respondents showed a reduction in psychoactive substance use since starting treatment, which was significantly more common in men (44% vs. 32%; χ^2^ = 8.6; *p* = 0.01; chi-square test).

Psychiatrists talked to patients about their psychoactive substance use before treatment in 75% of cases, but only during treatment in 39% of cases.

### Consumption of psychoactive substances

A summary of psychoactive substance use is described in Table 3. 64% of respondents drank alcohol, and 43% consumed psychoactive substance other than alcohol. No significant differences were found between genders in the use of alcohol or psychoactive substances (*p* > 0.05; chi-square test).

**Table 3:** Characteristics of psychoactive substance used among respondents.

		All	M	K	Other	*p* value M *vs* K (χ^2^)
	*n*	438	88	330	20	
	%	100%	20.1%	75.3%	4.6%	
Do you use alcohol?	Yes	63.5%	67.1%	62.4%	65.0%	NS
	No	36.5%	32.9%	37.6%	35.0%	
Do you use psychoactive substances other than alcohol (below)?	Yes	42.7%	46.6%	40.0%	70.0%	NS
	No	57.3%	53.4%	60.0%	30.0%	
How many psychoactive substances do you use?	Many	22.1%	29.5%	18.8%	45.0%	NS
	One	20.6%	17.0%	21.2%	25.0%	
	None	57.3%	53.4%	60.0%	30.0%	
THC	Yes	37.4%	44.3%	33.9%	65.0%	NS
	No	62.6%	55.7%	66.1%	35.0%	
MDMA	Yes	13.7%	20.5%	11.2%	25.0%	*p* = 0.022
	No	86.3%	79.5%	88.8%	75.0%	
Amphetamine/Methamphetamine	Yes	10.1%	11.4%	8.5%	30.0%	NS
	No	90.0%	88.6%	91.5%	70.0%	
Psylocybin	Yes	10.1%	12.5%	8.5%	25.0%	NS
	No	90.0%	87.5%	91.5%	75.0%	
Cocaine	Yes	5.5%	6.8%	4.8%	10.0%	NS
	No	94.5%	93.2%	95.2%	90.0%	
LSD	Yes	9.1%	10.2%	8.5%	15.0%	NS
	No	90.9%	89.8%	91.5%	85.0%	
Mephedrone	Yes	7.1%	11.4%	5.8%	10.0%	NS
	No	92.9%	88.6%	94.2%	90.0%	
Opioids	Yes	3.7%	8.0%	2.4%	5.0%	*p* = 0.013
	No	96.4%	92.0%	97.6%	95.0%	
Ketamine	Yes	3.4%	3.4%	3.0%	10.0%	NS
	No	96.6%	96.6%	97.0%	90.0%	
DMT	Yes	2.1%	1.1%	1.5%	15.0%	NS
	No	98.0%	98.9%	98.5%	85.0%	
2CB	Yes	0.7%	2.3%	0.3%	0.0%	NS
	No	99.3%	97.7%	99.7%	100.0%	
GHB/GBL	Yes	0.5%	0.0%	0.3%	5.0%	NS
	No	99.5%	100.0%	99.7%	95.0%	
Other	Yes	0.7%	0.0%	0.9%	0.0%	NS
	No	99.3%	100.0%	99.1%	100.0%	

NS, non significant; χ^2^, Chi-squared test.

The most frequently used psychoactive substances were again THC-containing products (37%), 3,4-methylenedioxymethamphetamine (MDMA) (14%), and amphetamine/methamphetamine and psilocybin (10% each). Males with ADHD were more likely than females to use MDMA (χ^2^ = 5.2; *p* = 0.022; chi-square test) and likewise opioids (χ^2^ = 6.1; *p* = 0.013; chi-square test). Otherwise, there were no gender differences.

### AUDIT and DUDIT scales

Table 4 presents data on the distribution of alcohol and drug abuse risk groups using the AUDIT and DUDIT scales. The mean score on the AUDITscale (0–40 points) was 6.8 ± 4.8 and on the DUDITscale (0–44 points) was 8.6 ± 6.8. There was no significant association between gender and scores on the AUDIT and DUDIT scales (Kruskal-Walis test, *p* > 0.05).

**Table 4:** Distribution of mean scores and groups with clinical significance in AUDIT and DUDIT scales among genders.

			All	M	K	Other	*p* value
		*n*	438	88	330	20	
		%	100%	20.1%	75.3%	4.6%	
No alcohol use			36.5%	33%	37.6%	35%	NS (χ)^2^
AUDIT 0-40pts	1	1–7 (low risk consumption)	42.7%	45.5%	41.5%	50%	
	2	8–14 (hazardous or harmful consumption)	16.2%	15.9%	16.7%	10%	
	3	≥15 (likelihood of alcohol dependence)	4.6%	5.7%	4.2%	5%	
		mean result AUDIT ± SD	6.8 ± 4.8	7.4 ± 5.0	6.7 ± 4.8	6.0 ± 4.0	NS (K-W t)
No drugs use			57.3%	53.4%	60%	30%	*p* < 0.0001(χ)^2^
DUDIT 0-44pts	1	below cut-off	4.3%	15.9%	1.2%	5%	
	2	M: ≥6 K:≥2 (drug-related problems)	36.8%	29.5%	37.6%	55%	
	3	≥25 (highly probable dependence)	1.6%	1.1%	1.2%	10%	
		mean result DUDIT ± SD	8.6 ± 6.8	9.3 ± 7.0	8.0 ± 6.4	12.0 ± 9.6	NS (K-W t)

NS, non significant; M-W U test, Mann Whitney U Test; χ^2^, Chi-squared test; KW test, Kruskal-Wallis H test; ^*^Significant difference between other vs. M and other vs. K; NS M vs. K.

16% of respondents are characterized by risky or harmful alcohol consumption, while 5% meet the criteria for the likelihood of alcohol dependence.

37% of respondents exhibit drug-related problems, according to DUDIT, while approximately 2% are highly likely to be drug dependent. A statistically significant gender difference is found regarding classification into risk groups for drug abuse based on the DUDIT scale (χ2 = 50.2; *p* < 0.00001; chi-square test).

An inverse relationship is observed between age and the amount of psychoactive substance taken equals the lower the age, the greater the amount of psychoactive substance taken (Spearman Rank’s; *R* = −0.12; *p* = 0.011). The earlier the age at ADHD diagnosis, the higher the DUDIT score (Spearman Rank’s; *R* = −0.18; *p* = 0.013). A similar relationship is observed between age of diagnosis and level of psychoactive substance use (Spearman Rank’s; *R* = −0.14; *p* = 0.002). No such correlation is observed for the AUDIT scale (Spearman Rank’s; *p* > 0.05).

A correlation is observed between the AUDIT scale score and the DUDIT scale (Spearman Rank’s; *R* = −0.2; *p* = 0.013).

Table 5 shows the distribution of AUDIT and DUDIT scores across ADHD types and by treatment outcome. There is no difference between ADHD types in terms of AUDIT, while there is a significant difference in terms of DUDIT. No significant difference was found between the groups with and without improvement after treatment in terms of AUDIT and DUDIT. Similarly, no significant difference was found between the groups at risk of abuse in relation to AUDIT and DUDIT (Kruskal-Walis test *p* > 0.05). A significant difference was found between the groups with and without drug treatment on both AUDIT and DUDIT (Mann–Whitney U test; AUDIT: *p* = 0.019/DUDIT: *p* = 0.0002).

**Table 5:** Distribution of mean scores on the AUDIT and DUDIT scales among ADHD types and according to improvement after medication.

		%	AUDIT	DUDIT
ADHD type	Do not know/subtype not specified	25.8%	6.6 ± 4.4	8.5 ± 7.5^*^
	Mixed type ADHD	34.9%	6.6 ± 5.2	7.1 ± 5.4^*^
	ADHD with predominantly attention deficit disorder	37.9%	7.1 ± 4.8	9.9 ± 7.4^*^
	ADHD with predominance of hyperactivity and impulsivity	1.4%	4.5 ± 0.7	9.3 ± 5.3^*^
Improvement about drugs	Not	27.3%	6.8 ± 5.2	9.6 ± 8.2
	Yes	72.7%	6.8 ± 4.7	8.4 ± 6.6
Has drug treatment ever been undertaken?	Not	94.1%	6.67 ± 4.72^*^	7.71 ± 5.98^*^
	Yes	5.9%	10.1 ± 6.16^*^	15.9 ± 8.86^*^

^*^p < 0.05.

### Linear regression analysis for the scales AUDIT and DUDIT

Univariate and multivariate linear regression analyses were performed for the AUDIT and DUDIT scales. In the univariate analysis for AUDIT, the variables age and rehabilitation history were found to be statistically significant. The variables from the univariate analysis with *p* < 0.05 and 2 clinically significant-gender and type of ADHD-were included in the multivariate analysis, and as a result, the only significant factor conditioning AUDIT was found to be rehab history. The results are presented in Table 6.

**Table 6:** Univariate and multivariate analysis for parameters affecting AUDIT score.

	Univariate analysis				Multivariate analysis			
	*β*	SE	*t*	*p*	*β*	SE	*t*	*p*
Sociodemographical								
Gender	0.71	0.71	0.99	0.323	0.47	0.92	0.52	0.607
Age	−0.06	0.04	−1.47	0.142				
Heterosexual/ Non-heterosexual	−0.60	0.64	−0.93	0.351				
Size of city of residence	−0.30	0.38	−0.79	0.431				
Level of education	−0.29	0.31	−0.92	0.358				
ADHD								
Type of ADHD	−0.42	0.72	−0.59	0.556	−0.43	0.72	−0.60	0.546
Age of ADHD diagnosis	−0.03	0.04	−0.94	0.347				
ADHD pharmacotherapy								
Methylphenidate	−0.72	0.68	−1.05	0.293				
Atomoxetin	1.04	0.98	1.06	0.289				
Duloxetin	−0.75	1.49	−0.50	0.615				
Amount of medicaments	−0.14	0.54	−0.26	0.793				
Improvement after pharmacotherapy	0.00	0.82	0.00	0.997				
Medicaments withdrawal	−0.78	1.33	−0.59	0.558				
Psychoactive substance								
Went through rehab	3.43	1.47	2.33	0.021^*^	3.71	1.80	2.06	0.041^*^
Change of psychoactive substance use after treatment	0.10	0.53	0.19	0.847				
Talk with doctor about psychoactive substance								
before treatment	−0.48	0.70	−0.69	0.492				
after treatment	−0.37	0.61	−0.60	0.547				

^*^p < 0.05.

In the univariate analysis for DUDIT, the variables ADHD type, age at ADHD diagnosis, use of methylphenidate, and rehab history were found to be statistically significant. Variables from the univariate analysis with *p* < 0.05 and 1 clinically significant-gender-were included in the multivariate analysis. In the multivariate model, 2 variables were found to be statistically significant-the type of ADHD and history of rehospitalization. The results are presented in Table 7.

**Table 7:** Univariate and multivariate analysis for parameters affecting DUDIT score.

	Univariate analysis				Multivariate analysis			
	*β*	SE	*t*	*p*	*β*	SE	*t*	*p*
Sociodemographical								
Gender	1.33	1.17	1.14	0.256	1.32	1.229	1.07	0.286
Age	−0.11	0.08	−1.34	0.183				
Heterosexual/ Non-heterosexual	−0.84	1.02	−0.83	0.410				
Size of city of residence	−1.24	0.71	−1.74	0.084				
Level of education	−0.85	0.53	−1.60	0.111				
ADHD								
Type of ADHD	−2.09	0.98	−2.14	0.034^*^	−2.09	0.957	−2.18	0.031^*^
Age of ADHD diagnosis	−0.15	0.07	−2.22	0.027^*^	−0.08	0.081	−0.94	0.348
ADHD pharmacotherapy								
Methylphenidate	−2.64	1.15	−2.29	0.023^*^	−1.79	1.230	−1.46	0.147
Atomoxetin	1.46	1.59	0.92	0.359				
Duloxetin	−1.86	2.37	−0.79	0.432				
Amount of medicaments	0.23	0.95	0.24	0.810				
Improvement after pharmacotherapy	−1.76	1.43	−1.23	0.222				
Medicaments withdrawal	2.99	2.36	1.26	0.208				
Psychoactive substance								
Went through rehab	8.21	1.86	4.42	<0.0001^*^	4.58	2.064	2.22	0.028^*^
Change of psychoactive substance use after treatment	−0.08	0.85	−0.10	0.922				
Talk with doctor about psychoactive substance								
Before treatment	−1.26	1.22	−1.03	0.303				
After treatment	0.11	1.00	0.11	0.914				

^*^p < 0.05.

## Discussion

Attention deficit hyperactivity disorder (ADHD) is a disorder that affects many areas of human life. Among other things, people with ADHD show a greater tendency to engage in risky behaviors, such as gambling, they tend to leave their jobs abruptly and spend their money recklessly, and they have a higher risk of physical injury or suicide in accidents (1). Meta-analyzes have also shown that people with ADHD are significantly more likely to use psychoactive substance also including alcohol and nicotine (20).

In a study we conducted, methylphenidate was found to be the most commonly used medication among people with ADHD in both males and females. This drug is also the most thoroughly studied and has a proven efficacy benefit: symptoms improve shortly after ingestion, and the effect lasts until metabolism in the body is complete (21). It also seems important to note that immediate-release methylphenidate is the only medication with proven efficacy in ADHD patients who use psychoactive substances (22).

A significant proportion of respondents to our survey indicated that substance abuse had decreased since treatment began, particularly in the male group, which appears to be important information in the context of conducting appropriate diagnostic testing and treatment engagement. The results of previous studies on the impact of appropriate treatment on reducing psychoactive substance use remain mixed. In part, it has been suggested that treatment use is neither protective nor beneficial for the development of psychoactive substance use disorders later in life (23). On the other hand, the literature also shows that the use of stimulants to treat ADHD in childhood significantly reduces the risk of developing dependence later in life (24). An interesting observation seems to be the fact that in a study investigating the effect of treatment on nicotine addiction, it was shown that patients were significantly less likely to use tobacco products after pharmacotherapy with stimulants (25). Studies showing improvement in both clinical symptoms and reduction in substance use during treatment with stimulants required the use of high doses of medication, which necessitates further research on the topic (26). For example, atomoxetine is generally considered a safe and well-tolerated drug, but it is important for physicians to be mindful of the potential cardiovascular adverse effects associated with its use to prevent cardiovascular complications, including the possibility of myocardial dysfunction (27). These tend to get worse with high doses.

The validated DUDIT scale shows that a significant percentage of respondents are highly likely to have a substance abuse problem, and a small percentage are highly likely to exhibit characteristics of substance dependence. It is worth noting that despite the fact that divergent results can be found in the literature, a significant number of meta-analyses indicate that childhood pharmacotherapy with simulants is associated with a reduction in the risk of later use of alcohol and psychoactive substances (28). Appropriate treatment of people with ADHD is important because it also helps to reduce the risk of injury in accidents, including by reducing the risk of turning to psychoactive substances, which also means a lower rate of injury (29).

Participants in our survey confirmed that they were most likely to use products containing THC, MDMA, amphetamine, and psilocybin. Men were significantly more likely than women to admit to using MDMA and opioids, whereas the differences for the other psychoactive substance were not significant. A 2016 study found that women are statistically more likely to become addicted to marijuana, as well as alcohol (30). It is important to note the prevalence of the extent of marijuana use in ADHD patients, as there are reports suggesting that cannabis abuse is genetically positively correlated with the occurrence of ADHD, schizophrenia, and major depressive episodes (31). One of the better studied substances among ADHD users is cocaine. It has been shown that one in four people struggling with attention deficit hyperactivity disorder use this substance, while 10% develop cocaine dependence (32). There are reports that patients with ADHD turn to marijuana on their own and use it to self-medicate, although there is no clinical evidence of its efficacy. This is a troubling phenomenon that needs to be investigated and monitored, as respondents in our study also reported THC as the substance they resorted to most often (33). In the context of individuals with co-occurring bipolar disorder and ADHD and substance abuse tendencies, it has been observed that the inclusion of non-stimulants in treatment may be more beneficial than taking stimulants, but their full therapeutic effects last longer (34).

On the AUDIT scale, we observed a tendency toward risky alcohol consumption among the respondents, but also some of the respondents met the criteria for alcohol dependence. Previous studies have concluded that a higher AUDIT score is significantly correlated not only with previously diagnosed ADHD, but also with comorbid schizophrenia and major depressive disorder (35). In addition, individuals who had attention deficit hyperactivity disorder and concurrent bipolar affective disorder showed an increased tendency to abuse alcohol compared to the healthy population (36).

In a study to determine the average age at which ADHD symptoms are diagnosed and appropriate diagnosis and treatment are provided, it was suggested that accurate and appropriate early selection of pharmacotherapy will help prevent the consequences associated with the disorder and reduce society’s costs for the complications of ADHD (37). This is an important observation, as our study demonstrated that individuals previously diagnosed with ADHD score higher on the DUDIT scale and have a higher propensity to take psychoactive substance. There is also a correlation between the AUDIT and DUDIT scales, which is also reflected in previous observations demonstrating that individuals with attention deficit hyperactivity disorder not only have an increased tendency to use psychoactive substances, but also develop symptoms of alcohol dependence syndrome (3).

No difference was found between ADHD types in respondents on the AUDIT and DUDIT scales. Again, the data are mixed, as over the years it has been shown that the different subtypes do not have a clear impact on substance abuse or that the complex ADHD subtype is associated with more severe effects of psychoactive substance use and more frequent help seeking compared to the predominantly inattentive or predominantly hyperactive/impulsive subtype (38).

It is crucial to highlight the fact that only 38.80% of physicians asked about psychoactive substance use during the treatment. It is important for physicians to discuss psychoactive substance use with their patients during treatment to ensure patient safety and effective medical management – by understanding a patient’s substance use, physicians can identify potential interactions and adjust treatment plans accordingly. Additionally, discussing substance use opens the door for early detection and intervention for substance use disorders, promoting holistic care and improved patient outcomes. It is of utmost importance to properly diagnose and recognize characteristics that may indicate addiction development in patients, as people burdened by mental illness who use psychoactive substance have the highest mortality rate among other diagnoses (39, 40).

## Conclusion

People with ADHD use psychoactive substances relatively frequently, and the most commonly used psychoactive substance include alcohol, marijuana, MDMA, amphetamine/methamphetamine, and psilocybin. In the study population, methylphenidate is the drug most commonly used by patients. After treatment with psychoactive substance, there is a decrease in attention deficit hyperactivity disorder symptoms in most respondents, especially in male patients. It is important to perform proper diagnostics in patients prone to psychoactive substance use and to proactively screen for symptoms of ADHD, as properly applied treatment can help reduce troublesome symptoms.

### Limitations

The survey was distributed online without identifying respondents, so it is not possible to assess long-term substance use in the context of reducing psychoactive substance use. Because of the way the survey was distributed, it was not possible to calculate response rates. Because the survey was conducted over the Internet, respondents’ answers may not have been entirely reliable, but this was unavoidable given the topic of the survey. There was no way to verify the truthfulness of the answers given. The survey was exploratory in nature, and the topic needs to be further researched, as it seems important to make an accurate diagnosis and choose reasonable treatment at a time when ADHD is becoming more widely known, in order to minimize the potential health, social, and economic consequences of an untreated disorder. Because the study was naturalistic, there was no hypothesis and study conducted without a hypothesis lacks a clear research direction, which can lead to unfocused data collection and analysis. Some of the limitations lay also in the statistical methods used, as univariate linear regression analysis is limited in capturing the complexities of real-world relationships involving multiple predictors and assumes a linear relationship between a single predictor and the dependent variable. Multivariate linear regression analysis assumes no multicollinearity between predictors, and if present, it can lead to unstable coefficient estimates and difficulties in interpreting the relationships accurately. Both univariate and multivariate linear regression analyses may not account for non-linear relationships, outliers, or other underlying factors that can affect the accuracy and reliability of the regression model.

## Data availability statement

The raw data supporting the conclusions of this article will be made available by the authors, without undue reservation.

## Ethics statement

Ethical review and approval was not required for the study on human participants in accordance with the local legislation and institutional requirements. Online written informed consent for participation was obtained from the participants.

## Author contributions

GW: conceptualization, methodology, visualization, investigation, data curation, and writing—reviewing and editing. IS and MS: software, investigation, data curation, and writing—original draft preparation. WW: supervision, data curation, and investigation. PG: supervision and funding acquisition. TG: visualization, data curation, investigation, and writing—reviewing and editing. All authors contributed to the article and approved the submitted version.

## Conflict of interest

GW received honoraria for lectures from: Angelini Pharma Polska and support for attending meetings from: Angelini Pharma Polska, Apotex Poland/Aurovitas Pharma Polska, Lek-AM. IS received support for attending meetings from: Lek-AM. TG received honoraria for lectures from: Valeant Polska, Lundbeck Poland, Apotex Poland/Aurovitas Pharma Polska, Celon Pharma and support for attending meetings from: Lundbeck Poland, EGIS, GL Pharma.

The remaining authors declare that the research was conducted in the absence of any commercial or financial relationships that could be construed as a potential conflict of interest.

## Publisher’s note

All claims expressed in this article are solely those of the authors and do not necessarily represent those of their affiliated organizations, or those of the publisher, the editors and the reviewers. Any product that may be evaluated in this article, or claim that may be made by its manufacturer, is not guaranteed or endorsed by the publisher.
